# CO_2_ Electrolysis Technologies: Bridging the Gap toward Scale-up and Commercialization

**DOI:** 10.1021/acsenergylett.4c00955

**Published:** 2024-08-09

**Authors:** Blanca Belsa, Lu Xia, F. Pelayo García de Arquer

**Affiliations:** † The Barcelona Institute of Science and Technology, ICFO - Institut de Ciències Fotòniques, Castelldefels, Barcelona 08860, Spain

## Abstract

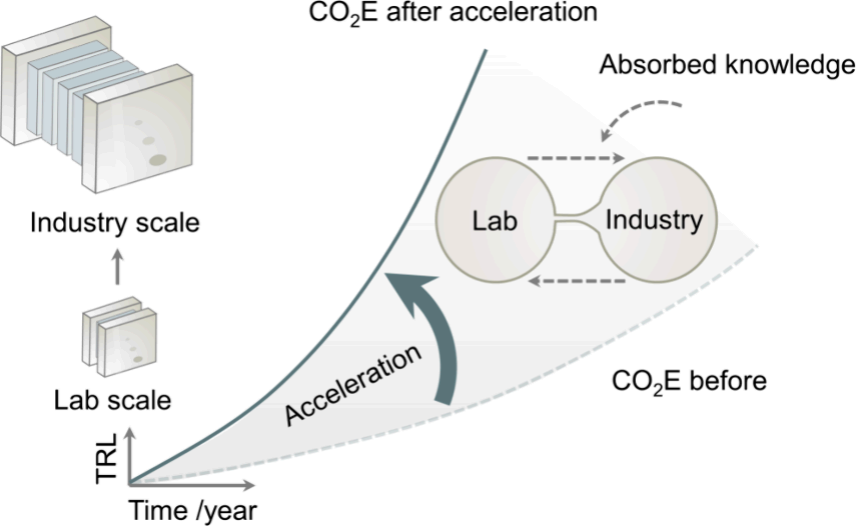

CO_2_ electroreduction (CO_2_E) converts CO_2_ into carbon-based fuels and chemical feedstocks that can be integrated into existing chemical processes. After decades of research, CO_2_E is approaching commercialization with several startups, pilot plants, and large initiatives targeting different products. Here, we analyze the global efforts in scaling up CO_2_E, addressing implementation challenges and proposing methods for acceleration. We present a comparative analysis of key performance indicators (KPIs) between laboratory and industrial settings and suggest a stepwise technoeconomic analysis (TEA) framework, supported by industrial data, exploiting interactions within the academic and industrial communities. We identify the lack of systems-oriented standardization and durability as the main bottlenecks slowing down progress in the lab-to-prototype-to-market pathway of CO_2_E technologies. Inspired by electrolysis and fuel cell technologies, we outline protocols to advance fundamental research and aid catalyst development progress in performance, upscaling, and technology readiness level of CO_2_E.

The intense reliance on fossil fuels with their associated greenhouse gas emissions has drastically accelerated global warming during the last decades. Fuels for heating and transport and chemical feedstocks for manufacturing, agriculture, and the general industry are together responsible for nearly 50% of global CO_2_ emissions.^1^ This has surged the need for a rapid transition toward sustainable energy harvesting, storage, and utilization.

The electrification of these industries using renewables (e.g., solar and wind) or low carbon footprint energy represents a viable pathway to decarbonize widespread global processes.

Batteries and hydrogen (H_2_) derived from water electrolysis are two promising decarbonizing vectors to enable this shift. The first stores and releases electricity on demand and is indispensable to consumer electronics and electric cars. The development of green H_2_ produced from renewable sources, as opposed to fossil fuel-based processes, is gaining traction as an energy and chemical vector.^2^

Carbon capture and utilization technologies offer an additional decarbonizing path that recycles atmospheric and industrial CO_2_ into widely used carbon-based chemicals, which are today obtained from fossil fuels (Figure 1a).^3^ Under certain conditions, these technologies offer a path toward full carbon neutrality (net zero) and even negative emissions.^4^

**Figure 1:**
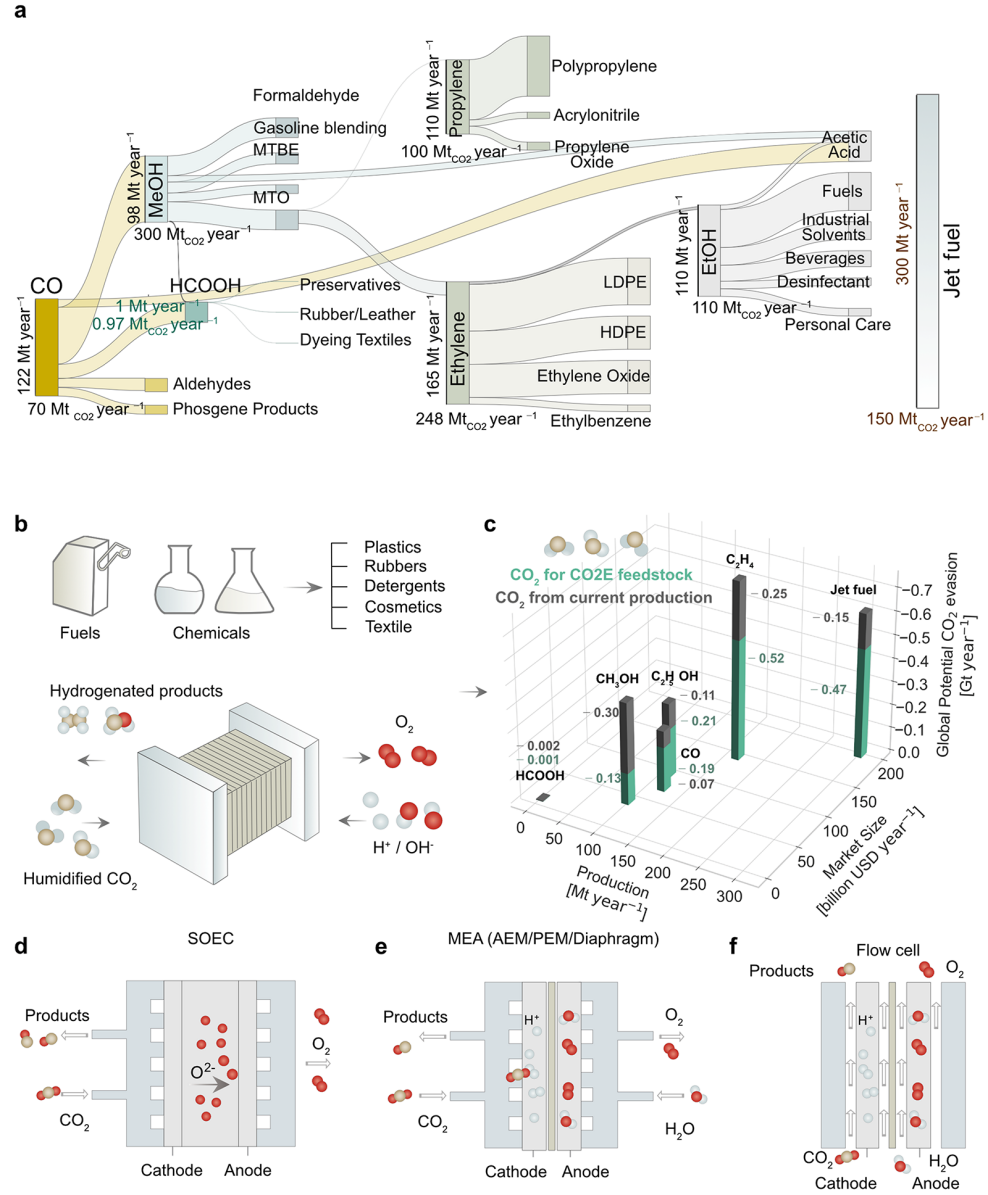
CO_2_ electrolysis (CO_2_E) as an alternative to fossil fuels for the production of the most common petrochemicals and fuels. (a) The production of chemicals and fuels is heavily reliant on fossil fuels. Their current production requires a substantial amount of energy and emits a significant amount of CO_2_ every year. The Sankey diagram (top) shows the current production schemes for the primary petrochemicals and their applications. Each box’s height denotes the yearly total production volume, while its width displays the CO_2_ emissions from its production. (b) CO_2_E is one potential alternative for producing these compounds in a sustainable manner by employing CO_2_ waste, water, and power at low temperatures and pressures. (c) CO_2_ emissions from the manufacture of such compounds, as well as CO_2_ from the atmosphere, might be collected and used as feedstock. The bar plot estimates the global CO_2_ evasion potential if CO_2_E were to totally replace fossil fuels. The *x*-axis shows the annual production in Mt (million of metric tonnes), the *y*-axis the market size in billion USD per year, and the *z*-axis the total amount of CO_2_ emissions per year that can be reduced by providing all actual production by means of CO_2_E. The annual global CO_2_ reduction potential comprises the CO_2_ needed as feedstock (blue-green) and the CO_2_ emissions avoided (gray) if current industrial manufacturing processes were displaced (Section S1). Classification of CO_2_ electrolysis technologies: (d) SOEC, (e) zero-gap MEA based on AEM, PEM, and diaphragm, and (f) flow cell.

Some carbon utilization examples include hydrogenation,^5^ algae production,^6^ plasma catalysis,^7^ photocatalysis,^8^ and CO_2_ electrolysis (CO_2_E).^9^ While some of these innovations are nearing commercialization, others are still at a lower technology readiness level (TRL) (Table S5).^10^

Akin to water electrolysis, CO_2_E converts CO_2_ molecules into small carbon-based molecules (C_*x*_H_*y*_O_*z*_), using electric energy to break CO_2_ and water into fragments and coupling them over a cathode catalyst (Figure 1b). The process is typically complemented by the oxidation of water to O_2_ to close the circuit. The resulting C_*x*_H_*y*_O_*z*_ molecules can serve as clean fuels (e.g., ethanol), be integrated into current supply chains for the production of global chemicals and materials, or be used for long-term energy storage. Molecules with increasing numbers of carbons, referred to as C_1_, C_2_, etc., generally have greater economic and energy value.^9^

While CO_2_E technologies are at a lower TRL compared to water electrolysis, there are several initiatives nearing commercialization and large pilots at increasing scales. The pressing need to deploy decarbonizing technologies raises the question of how we could accelerate the scale-up transition of CO_2_E from lab to market, beyond circumstantial factors such as market demand, regulatory frameworks, and funding. Such a chicken and egg dilemma in funding new technology is a common challenge, particularly in cases that demand large investments and implementation at scale.^11^

Here, we scrutinize global efforts to scale up CO_2_E, including examples of startups and multinational corporations, and their value proposition. We focus on the potential use of CO_2_E for producing chemicals and jet fuel, while recognizing the significant market for CO_2_-derived fuels in diesel and Otto engines. We offer an overview of laboratory and industrial performance metrics and discuss strategies to bridge the gap between research and development at these scales, seeking to accelerate the maturity and deployment of CO_2_E. We discuss the critical role of promoting interdisciplinary interactions and knowledge exchange from these different settings, and we propose strategies and protocols adapted from electrolysis and fuel cell technologies to overcome these barriers. We conclude by offering a broad vision of the future prospects of CO_2_E within these growing electron-driven technologies.

## Toward a Circular Economy: Energy and Petrochemical Industry Transformation

The energy and petrochemical industries rely on fossil fuels, primarily oil and natural gas, to produce various products including plastics, fibbers, coatings, fertilizers, and fuels, among others. Some key chemicals, such as syngas [i.e., carbon monoxide (CO) + H_2_], methane, methanol, ethanol, ethylene, and propylene, play a crucial role as building blocks in these processes (Figure 1a, Table S1). These are produced through different thermochemical processes, including bottom-up (Fischer–Tropsch, reverse water–gas shift reaction, olefination) and top-down (e.g., fractional distillation for gasolines) processes, at different rates up to hundreds of Mt year^–1^.

Among these, ethylene and jet fuels stand out as the largest CO_2_ emission contributors (Table S2). Ethylene has the largest production volume (∼165 Mt year^–1^), surpassing that of any other organic compound.^12^ Traditional cracking methods to produce ethylene result in significant CO_2_ emissions, contributing to over 250 Mt year^–1^.^13^ The challenges associated in the electrification of aviation transport make jet fuels a difficult-to-displace, crucial resource for aviation (produced at a rate of 300 Mt year^–1^)^14^ with a large carbon footprint.^15^

CO_2_E offers a path to producing some of these chemicals (Table S3, common reactions) using captured CO_2_ as a feedstock, thus potentially enabling a large CO_2_ evasion (Figure 1c). We estimated the maximum global CO_2_ evasion potential if CO_2_E were to totally replace fossil fuels in these processes (Figure 1c). This estimation is an upper bound considering two factors: the global CO_2_ emissions avoided by discontinuing current industrial output and the CO_2_ consumed as a feedstock through CO_2_E (Section S1). Among these products, ethylene shows the highest CO_2_ evasion potential of over 0.7 Gt year^–1^, followed by jet fuel (>0.6 Gt year^–1^), methanol (>0.4 Gt year^–1^), and ethanol (>0.3 Gt year^–1^) (Table S2). In total, CO_2_E could address nearly ∼2 Gt_CO_2__ year^–1^ of global emissions. This combined impact would account for approximately 5% of global anthropogenic CO_2_ emissions. CO_2_E could contribute to a fraction of this within the broader spectrum of carbon mitigation strategies.

CO_2_ electrolyzers are broadly classified into two main types: (i) high-temperature electrolysis, which includes solid oxide electrolyzers (SOECs) employing ceramic electrolytes at elevated temperatures (Figure 1d), and (ii) low-temperature electrolysis, which encompasses zero-gap membrane electrode assemblies (MEAs) based on polymer electrolyte membranes (Figure 1e), as well as flow cells utilizing catholyte and anolyte compartments separated by ion-selective membranes (Figure 1f). The specific configuration of each type influences material selection and system components.

The performance of CO_2_E systems is assessed using different indicators with more relevance at the laboratory (faradaic efficiency, partial current density, energy efficiency, carbon efficiency, etc.) and industrial settings (product purity, productivity, capacity, and reliability) (Table 1).

**Table 1:** Figures of Merit (FoM), Key Performance Indicators (KPIs), and Their Impact

FoM	definition	impacts
faradaic efficiency (FE)	FE is a measure of the selectivity in an electrochemical process. It is defined as the ratio of moles of product to the amount that could be produced from the total charge passed, expressed as a fraction or a percent:	energy efficiency, product separation, operational expenditure (OpEx, see below)
	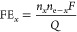	
	where *n*_*x*_ is the amount of product *x* (mol), *n*_e__–*x*_ is the number of electrons to make *x* from CO_2_/H_2_O, *F* is the Faraday constant (96 485 C mol^–1^), and *Q* is the total charge passed.	
partial current density (*J*_*x*_)	The partial current density relates selectivity (FE_*x*_) to CO_2_E reaction rate:	productivity, product concentration, capital expenditure (CapEx, see below)
	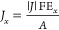	
	where *J* is the current passed by the electrode and *A* is the area of the electrode.	
energy efficiency (EE_*x*_)/energy consumption	The ratio of the energy input for CO_2_R to the energy content of the resulting products (output/input):	OpEx
	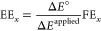	
	where Δ*E*° is the equilibrium full cell potential (Δ*E*° = *E*°_CO_2_/*x*_ – *E*°_water oxidation_), Δ*E*^applied^ is the applied full cell voltage, and FE_*x*_ is the average faradaic efficiency for the specific product.	
	EE can also be referred to as the energy input per unit mass product (kWh/kg or MWh/t).	
carbon efficiency (carbon eff.)	The ratio of C-based molecules rate to input CO_2_ rate: 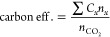	product concentration, product separation, process efficiency, carbon savings
	where *C*_*x*_ is the number of carbons in product *x*, *n*_*x*_ is the number of moles of product *x* produced, and *n*_CO_2__ the number of moles of CO_2_ that entered the system.	
product purity	The degree of absence of impurities or contaminants in a substance (%):	separation costs; process efficiency; product quality, safety, and reliability
	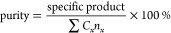	
productivity/capacity	Capacity is the maximum output a system can handle (t/day), while productivity measures the efficiency of resource utilization to achieve that output (MWh/t).	CapEx, economic viability
stability	The duration over which a performance metric (typ. FE, partial current, or voltage) is retained within a given interval.	overall viability
CapEx	Long-term investments made in the electrolytic system and plant.	return of investment, economic viability
OpEx	Regular costs associated with maintaining and running the plant.	running costs, economic viability
reliability	The probability that the system will perform its intended function adequately for a specified period of time or will operate in a defined environment without failure.	overall viability

## Assessment of Existing Efforts toward CO_2_E Scale-up

CO_2_E performance has improved over the last years, especially toward CO, formate, and ethylene. These can be produced at high selectivity and rates (1 A cm^–2^), prompting efforts toward scale-up and industrialization.^16^ The selective generation of other relevant products such as methane and ethanol, and especially methanol and others, is still challenging at increasing current densities (>0.2 A cm^–2^). Broadly, these processes still lack sufficient stability, carbon utilization, and energy efficiency, based on reported lab-scale data. This study aims to draw pathways to accelerate progress in these KPIs as CO_2_E technologies scale up, fostering closer industry–lab cooperation and adopting learnings from more mature sister technologies (e.g., water electrolysis, fuel cells, and batteries), and initial pilot CO_2_E endeavors.

**Figure 2:**
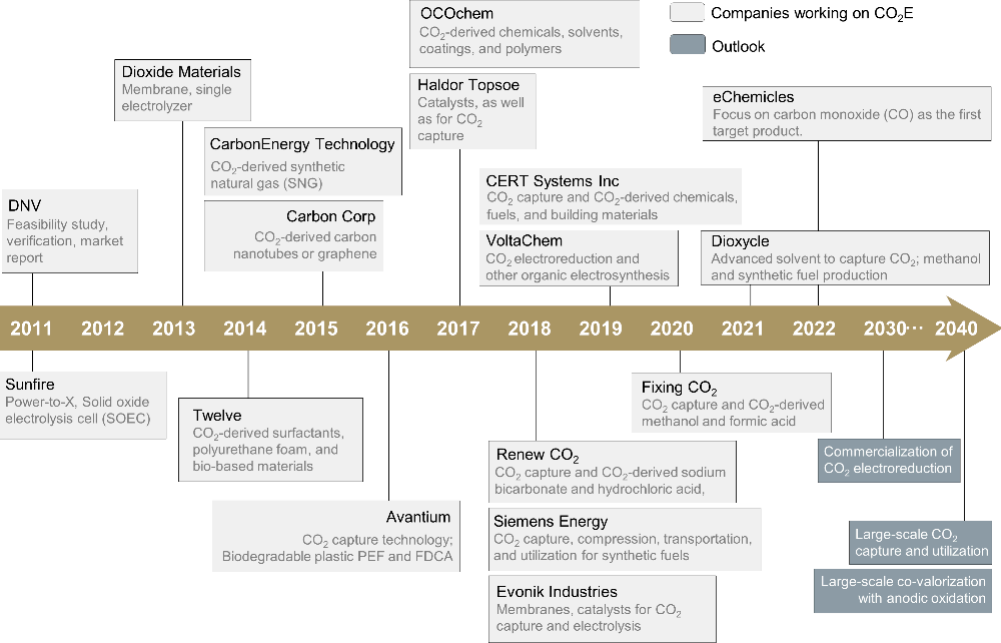
Timeline of existing efforts toward CO_2_E scale-up. The main companies working on CO_2_E are presented here, ordered by the year where they first reported significant efforts in CO_2_E for established companies or the founding year for startup companies. Examples are provided for firms such as Twelve, Dioxide Materials, Siemens Energy, CERT Systems, and eChemicles (from left to right).

**Figure 3:**
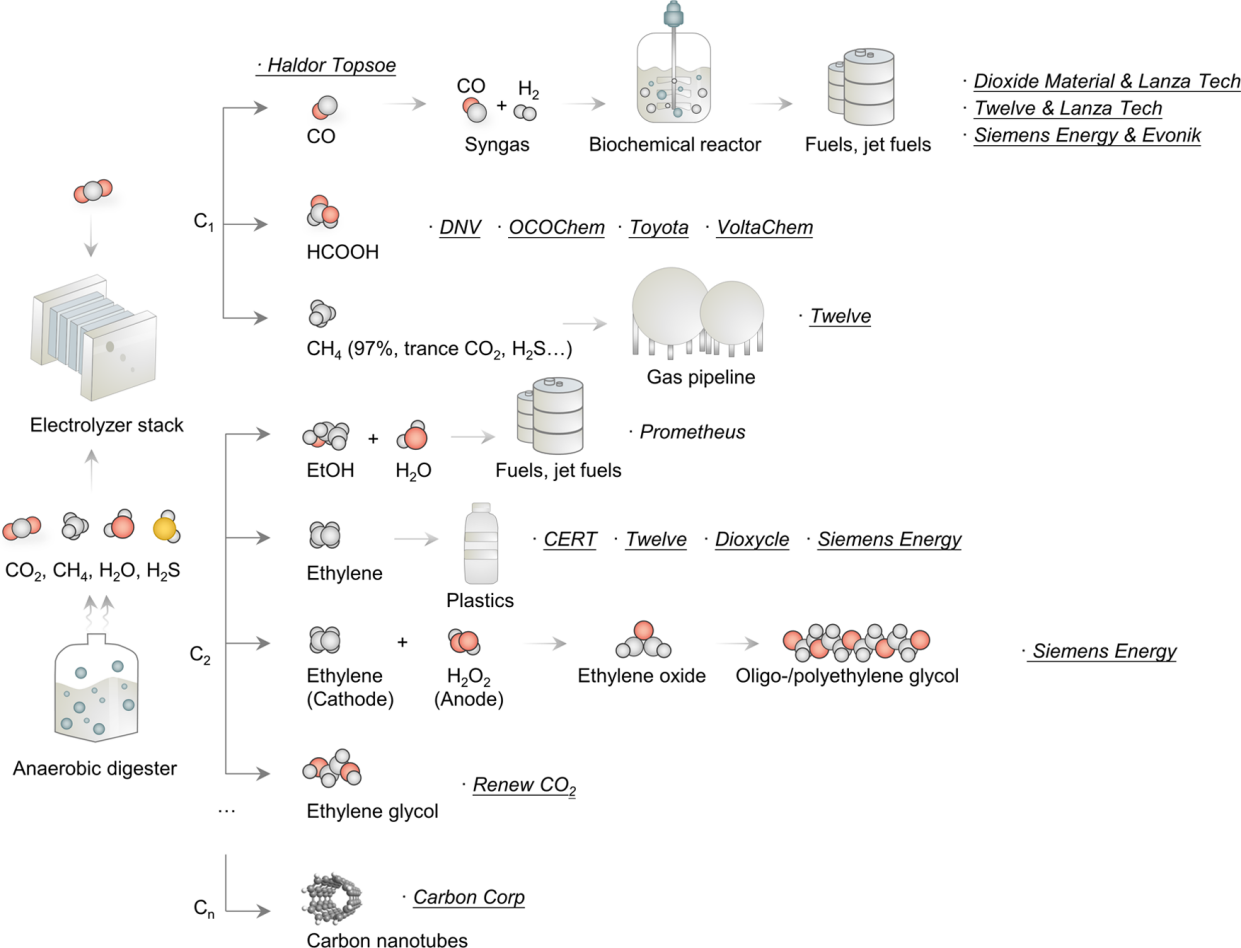
Summary of the different strategies and target products aimed at by each company on its path to large-scale CO_2_E. CO_2_ (CO_2_:CH_4_ 50:50) is fed into the electrolyzer as feedstock, resulting in (from top to bottom) CO or a CO/H_2_ mixture known as syngas that may be coupled to a biochemical reactor to produce fuels and jet fuels, HCOOH, (methane that may be introduced directly into the gas pipeline), ethanol to be used as fuel, ethylene for the production of plastics, ethylene glycol, or carbon nanotubes, among others.

Several pilot-scale CO_2_E systems have been, and are currently being, developed by small startups and larger enterprises since the 2010s (Figure 2, Table 2). Initial efforts focused on the production of carbon monoxide and formic acid. This was followed by ethylene and ethanol in the later years. Some initiatives have also reported the production of methane, oxalic acid, and higher end-products such as carbon nanotubes and jet fuels (Figure 3).

**Table 2:** Products, Strategies, and Highlights of CO_2_E Initiatives^a^

company	country	year	product	strategy	highlights
DNV	NO	2011	formic acid/formate	tin on porous carbon as the cathode; ion exchange membrane	high selectivity and efficiency; formic acid as a final product and a storage medium for H_2_ or CO
Sunfire	DE	2011	carbon monoxide, synthetic natural gas (SNG)	SOEC	syngas production rate: 750 Nm^3^ h^–1^; power consumption: 3.85 kWh/Nm^3^; PGM-free materials
Dioxide Materials	USA	2013	carbon monoxide	anion exchange membranes	>500 mA cm^–2^ at 3 V; FE > 95%; voltage increase: 6 μV/h; lifetime: 4 years; stability: 6 months
Twelve	USA	2014	carbon monoxide, methane, ethylene	catalyst design; PEM electrolysis; modular system	modular system that can scale to any need; reactor system designed for seamless integration
Carbon Energy Technology	CN	2015	SNG, synthetic oil, green methanol	catalyst; membrane electrode; electrolysis reactor	integrated direct air carbon capture with a second technology converting CO_2_ into fuels
Carbon Corp	USA	2015	carbon nanotubes or graphene	high-yield electrolysis in molten salts	converting CO_2_ into graphitic materials
Avantium	NL	2016	formate, oxalic acid, glycolic acid	CO_2_ electrolyzer in tandem	converting oxalic acid into glycolic acid; blending glycolic acid with lactic acid to produce polylactic-*co*-glycolic acid (PLGA)
Haldor Topsoe	DK	2017	carbon monoxide	SOEC	various sizes and purities, up to 99.999% (grade 5.0); hazard-free handling during cylinder or trailer exchange
OCOchem	USA	2017	formate	proprietary modular stack called the Carbon Flux Electrolyzer	operating at room temperature and pressure; abundant tin metal serves as the catalyst; formate products with high purity
RenewCO_2_	USA	2018	monoethylene glycol (MEG)	selective catalyst design; ion exchange membrane; electrodeionization process	patented catalyst produces the monomer monoethylene glycol (MEG) with high selectivity; focus on optimizing and scaling up the CO_2_ to MEG reaction; primary use of MEG is in polyester manufacturing
Siemens Energy	DE	2018	carbon monoxide, ethylene	PEM electrolyzer; flow cell; preparation of GDEs	stack of 10 300 cm^2^ electrolysis cells: 25 kW for CO_2_ to CO; this, combined with hydrogen, provides the primary nutrients necessary for the bacteria in Evonik’s bioreactor to produce butanol and hexanol
Evonik Industries	DE	2018	butanol, hexanol	bioreactor for syngas upgrade to alcohols	bioreactors to generate butanol and hexanol; potential for producing other chemicals, depending on the bacterial strain and conditions
CERT Systems/U of T	CAD	2019	ethylene	MEA; catalyst design	converting CO_2_ into ethylene; five stacks with 10 cells each (800 cm^2^ area) with a projected capacity of 100 kg/day of CO_2_ into C_2_H_4_ and 2400 h cumulative operation
VoltaChem	NL	2019	carbon monoxide, formic acid, ethylene	low-temperature electrolyzers; SOEC; plasma technology	paired electrosynthesis; CO_2_ conversion into CO (cathode) coupled with 1,2-propanediol oxidation to lactic acid (anode); currently developing stack reactors with 15 cells (∼0.5 m^2^)
Fixing CO_2_	USA	2020	carbon monoxide	novel catalyst for CO_2_ to CO	patented and licensed inexpensive novel catalyst for CO_2_ to CO with 99% selectivity
Dioxycle	FR	2021	carbon monoxide, ethylene, synthetic fuel production	design its own catalytic cores with special metal alloys	novel membrane electrode assemblies for CO_2_ electrolyzers
eChemicles	HU	2022	carbon monoxide	novel catalysts; electrode assemblies; SolarCO_2_Value technology	first 2500 cm^2^ cell ready in October 2023

a Abbreviations: PEF, polyethylene furanoate; MEG, monoethylene glycol; FDCA, furandicarboxylic acid.

### CO_2_ to CO

**Figure 4:**
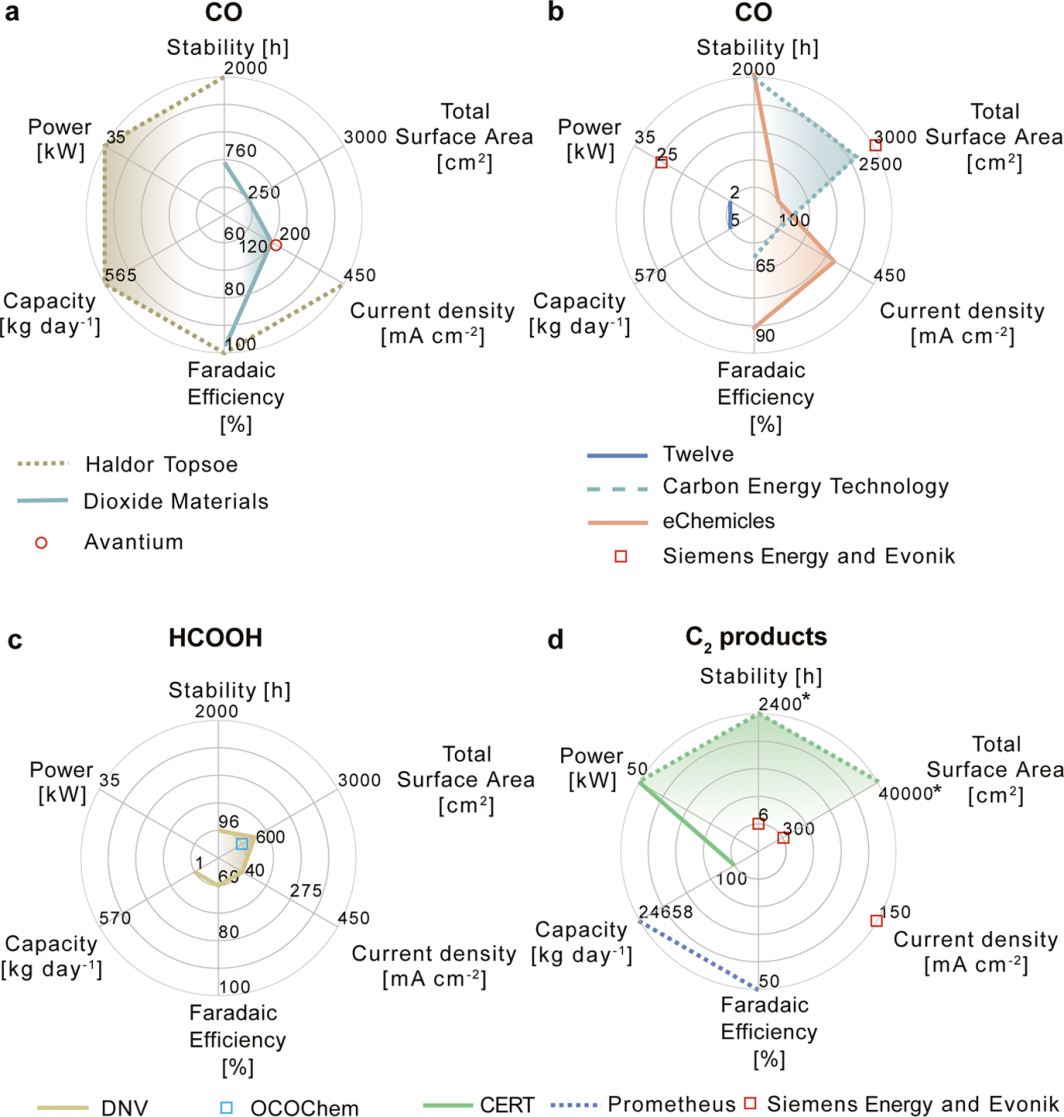
Reported performance metrics for different companies and end-products. The radar plots show the stability, total surface area, current density, FE, capacity, and power for each company technology and different target products: (a, b) carbon monoxide (CO), (c) formic acid/formate (HCOOH), and (d) C_2_ products such as ethanol (C_2_H_5_OH) and ethylene (C_2_H_4_). The dashed line in CERT Systems indicates cumulative operation (*). See Table S4 for more data and sources.

CO_2_E to CO initiatives, spanning high- and low-temperature electrolysis, are the ones closest to commercialization.^17^ Sunfire^18^ initiated early efforts of high-temperature SOEC technology and is now applying it to produce syngas (CO + H_2_) with a generation rate of 750 Nm^3^ h^–1^. Haldor Topsoe launched the first commercial SOEC system,^19^ demonstrating selective CO production (∼100% FE) and 2000 h stability at 0.45 A cm^–2^ (12 Nm^3^ h^–1^ production rate) (Figure 4a).^20^ Larger plants (96 Nm^3^ h^–1^ of CO) are under construction,^21^ with plans for modular electrolysis up to 2000 Nm^3^ h^–1^ by 2029.^22,23^

Dioxide Materials reported low-temperature CO_2_E by a 5 cm^2^ electrolyzer, which produces CO based on anion exchange membranes (AEMs) with minimized degradation after 4000 h.^24^ Additionally, a 250 cm^2^ CO_2_ electrolyzer was demonstrated, operating at 0.12 A cm^–2^ with ∼760 h stability at 2.8–3.0 V and an FE of 98% (Figure 4a, Table S4).^24^

Twelve (formerly Obtainium, later Opus 12) reported a 2 kW electrolyzer in 2019,^25^ converting 2 kg of CO_2_/day (Figure 4b, Table S4). This has been scaled up into a 50 kW unit (350 kg/day), finally targeting 1 MW electrolyzers (5 t/day).^25,26^

Other initiatives include Avantium,^27^ planning to operate a pilot plant in 2024 and enter the commercial stage in 2028;^28^ Siemens Energy,^29^ reporting CO_2_ to CO conversion at the lab scale (10 cm^2^) in 2018 with ∼70% FE at current densities up to 0.3 A cm^–2^ for over 1200 h; and VoltaChem,^30^ focusing on the generation of CO and HCOOH under their Power-2-Chemicals program (Figure 3).^31^

eChemicles specializes in low-temperature electrochemical CO_2_E, focusing on the conversion of CO_2_ into synthetic chemicals, starting with CO and subsequently expanding into diverse chemical markets, including plastics.^32^ They have recently reported stable operation during 2000 h with >90% FE at 300 mA cm^–2^ toward CO in an 8 cm^2^ cell (Figure 4b).^33^

Carbon Energy Technology showcased a stack with a 2500 cm^2^ area for CO_2_ electroreduction into syngas, with a CO selectivity ranging from 30% to 95%. The stability at 100 mA cm^–2^ was recorded as 2000 h with a cell voltage of 2.7–3.0 V (Figure 4b).^34^

### CO_2_ to HCOOH

DNV initiated the ECFORM project^35^ with a semipilot-scale reactor targeting formate (Figure 3). Their 2011 reactor (600 cm^2^) converted ∼1 kg of CO_2_/day to HCOOH with an FE of 60% (Figure 4c, Table S4), with stability progress from an initial 4 day operation^36^ to an FE over 75% at 0.15 A cm^–2^ sustained over an 8 day period.^37^

OCOchem (USA, 2019), reported HCOOH production from CO_2_E in 2017, achieving 2 kg of KCOOH with a small unit operating for several weeks (Figure 3).^38^

Others include VoltaChem through the project Power-2-HCOOH,^39^ aimed to deliver an electrochemical reactor producing small-scale HCOOH by the end of 2017,^40^ and Twence with a project expanding on Power-2-HCOOH focusing on scaling up.^41^

### CO_2_ to Methane

Twelve and SoCalGas developed a method to convert CO_2_ in raw biogas to methane (CH_4_) in a single electrochemical step. Biogas, a blend of CO_2_ and methane (CH_4_), is utilized to create synthetic natural gas. Twelve intends to convert the remaining CO_2_ waste to >97% pure CH_4_, doubling the production (Figure 3).^26,42^

### CO_2_ to Ethylene

Siemens Energy and partners in the CO_2_EXIDE project aimed at a paired CO_2_E system for simultaneous cathodic CO_2_E to ethylene (C_2_H_4_) and anodic H_2_O oxidation to hydrogen peroxide (H_2_O_2_), then coupled and upgraded to ethylene oxide and oligo-/polyethylene glycol (PEG) (Figure 3).^43^ The Siemens Energy laboratory also tested a 25 cm^2^ PEM cell and integrated it in a stack with a total area of 300 cm^2^, demonstrating operation at 0.15 A cm^–2^ and 6 h stability (Figure 4d, Table S4).

CERT Systems/University of Toronto (Canada, 2020) has begun scaling up CO_2_E into ethylene stemming from the Carbon XPRIZE challenge (Figure 3). The scale-up unit comprised five stacks with 10 cells 800 cm^2^ in area each, with a projected capacity to convert 100 kg of CO_2_/day into C_2_H_4_ and a cumulative operation of 2400 h (Figure 4d, Table S4).^44−46^ Drawing upon these pilot-scale CO_2_ electrolysis data, scale-dependent technoeconomic analysis (TEA) has been developed to bridge the gap between laboratory research and industrial implementations.^46,47^

Dioxycle is actively developing electrolyzer technologies to convert CO_2_ into small molecules, including CO, HCOOH, C_2_H_4_, etc.^48,49^

### CO_2_ to Other Chemicals

RenewCO_2_ uses CO_2_ and water to generate mostly monoethylene glycol (MEG) (Figure 3), methylglyoxal, and furandiol. RenewCO_2_ intends to unveil a commercial shipping container-sized solution capable of treating 3 tons of CO_2_ every day by 2025.^50,51^

Carbon Corp,^52^ another Carbon XPRIZE finalist (Figure 3), uses captured CO_2_ (from the environment or flue gas, by Carbon Corp) to realize carbon nanotubes (CNTs).^53,54^

### Tandem Conversion and Large Pilots toward High Energy Density Fuels

The integration of different CO_2_E strategies with additional upstream/downstream technologies opens a path to the generation of higher energy density fuels (e-jets) (Figure 3).

Prometheus proposes to convert CO_2_ into ethanol as a precursor to synthesize larger molecules through a carbon nanotube filtering method. In January 2021, they have reported a large, commercial-scale, electrochemical stack.^55−57^

Sunfire, together with Climeworks, Ineratec, and the Karlsruhe Institute of Technology, collaborated on the Kopernikus Power-to-X (P2X) project, combining four separate processes integrated in a compact plant.^58^ P2X intends to construct a unit capable of producing 200 L of synthetic fuel per day by 2022.^58,59^

Dioxide Materials, under the ARPA-E scale-up program, teamed up with Shell and LanzaTech to integrate a CO_2_ electrolyzer with microbial gas fermentation at the pilot scale to produce fuels and chemicals.^60^

Similarly, Siemens Energy connected their CO_2_ electrolyzer to a fermentation unit from Evonik, where the syngas produced from the CO_2_E was efficiently transformed into butanol and hexanol with an FE of ∼100%.^61^ Siemens Energy with Evonik are developing a pilot test of a stack of 10 300 cm^2^ electrolysis cells with a total output of roughly 25 kW in the Rheticus project (Figure 4b, Table S4).

VoltaChem validated their technology in a semicontinuous flow reactor,^62^ exploring CO_2_ conversion into CO coupled with 1,2-propanediol oxidation to lactic acid.^63^ They are currently developing stack reactors with 15 cells (∼0.5 m^2^).^64^

Avantium and partners are upscaling CO_2_ conversion pathways through a prepilot scale in the H2020 SPIRE project OCEAN, aiming to convert 250 g of CO_2_/h into formate with a 40 000 cm^2^ electrochemical stack operating at current densities >0.15 A cm^–2^. The formate obtained will be converted to oxalate, serving as an intermediate for producing higher-value products like glyoxylic acid and larger-volume manufacturing chemicals such as ethylene glycol.

## Key Performance Indicators (KPIs) and Upscaling Protocols

The industrialization of CO_2_E requires the standardization of KPIs and scalable protocols, together with a rigorous development of TEA and life-cycle assessment (LCA) models that combine both laboratory and industrial data inputs.^65^ Establishing a feedback loop between laboratory and industry efforts would be crucial to accelerate the deployment of CO_2_E technologies.^66^

### Different KPIs in Laboratory and Industry Settings

**Figure 5:**
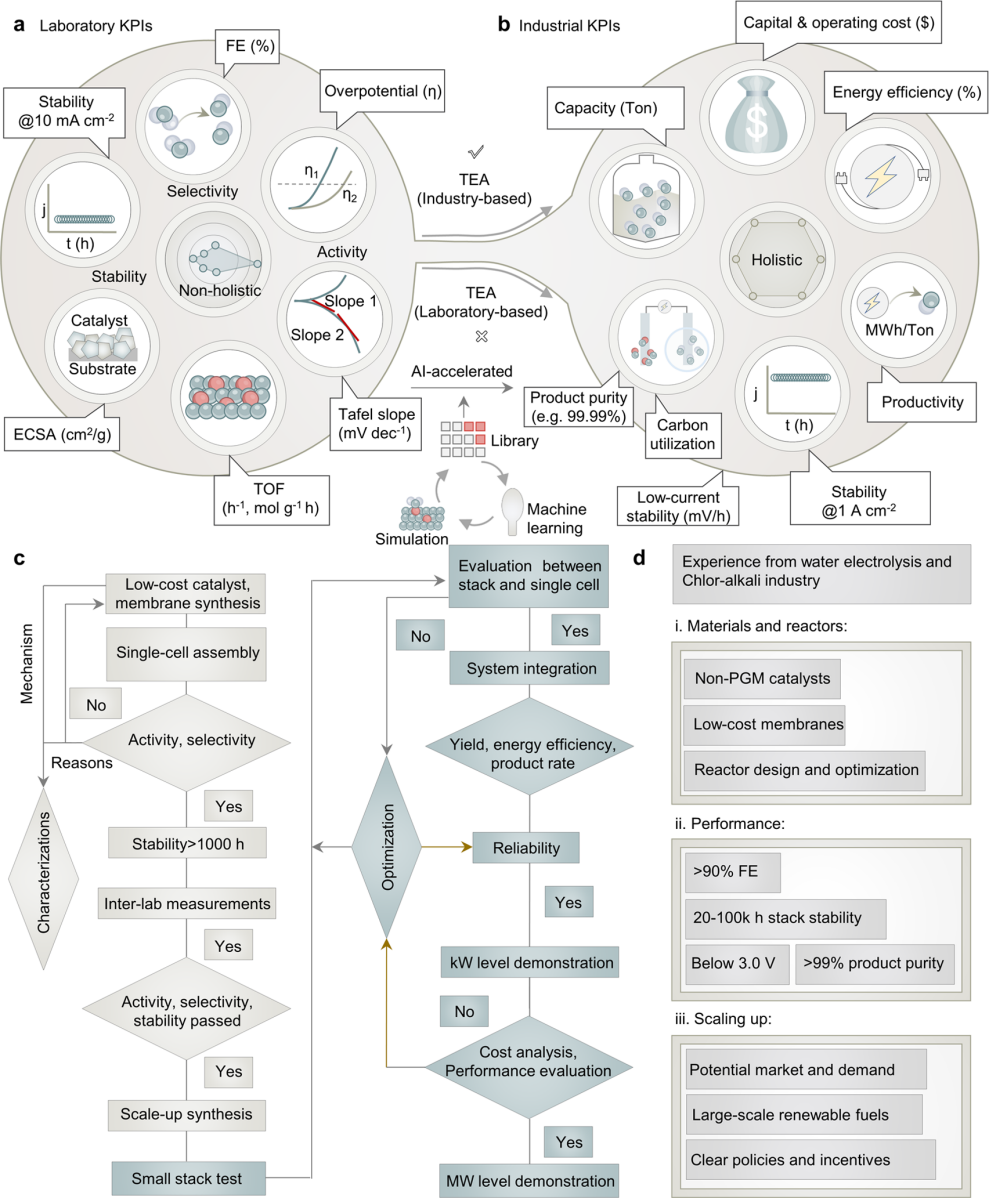
Key performance indicators (KPIs) and the protocol for CO_2_E. (a, b) Bridging laboratory and industrial KPIs using upscaling-based TEA to accelerate stepwise commercialization. (c) A typical example from laboratory low-cost catalyst and membrane synthesis to large-scale demonstration, inspired from water electrolysis technologies. (d) Experience for materials and reactor design, performance requirements, and upscaling from water electrolysis and chlor-alkali industries.

The KPI focus of laboratories and industry varies significantly. Laboratories typically focus on a subset of specific KPIs and related physical parameters from mechanistic studies (Figure 5a).^66^ Certain KPIs, such as degradation rate and stability, increasingly explored in lab settings, are also critical for industrial applications to reliability prospects on industrial scales.

Conversely, industry pursues advances over combined KPIs to ensure ultimate economic viability, primarily concerning production outcomes such as production yield, voltage efficiency, and durability (Figure 5b).^67,68^

Bridging the gap between fundamental research and industrialization requires effective methods that align research directions with the main pressing challenges toward the end application in a holistic approach, i.e., not relying on the improvement of individual KPIs at the expense of others.^8^ The significance of adopting a holistic approach in KPI reporting through their improvement is crucial, yet it is something not often implemented in academic circles. This will enable a more cohesive technology evaluation and prospect assessment.

In this context, the role of well-informed TEA and LCA models is critical.^69^ Currently, common models often rely on sparse experimental data on individual metrics from single/several laboratories, which are augmented by theoretical models to forecast the economic and technical behavior when scaled up.^70^

Lab-to-lab performance reproducibility and reliability is crucial to validate these figures, and formal statistical analysis should be considered to improve model predictive accuracy, including confidence intervals for each parameter.^71^

It is advisable to conduct TEA and scale-up concurrently, iteratively refining model predictions through the gradual collection of real-time scaled-up data.^66^ This feedback loop would allow a research focus that optimized scale-up processes based on economic and technical considerations.

Another important aspect is reflecting realistic and accurate self-consistent parameters in TEA models. Examples include using the right capacity factors if using renewables. These are often low, limited by the intermittency of renewable energy sources and operational constraints.^72^ We suggest using industrial data from existing water electrolysis operations as a realistic benchmark in TEA for CO_2_E, ensuring the analysis is both practical and grounded in current technological capabilities.

From a process perspective, the strong impact of CO_2_E upstream- and downstream-related technologies in final costs and environmental impact limits the accuracy of these analyses.^66^ Assuming cost and performance metrics from various technologies may not accurately reflect CO_2_E processes due to their unique operational parameters. Access to detailed data from small pilots and open databases for large projects could help address these discrepancies.

Well-defined protocols in fields like water electrolysis and fuel cells are essential for ensuring reproducibility and accelerating the transition from lab experiments to industrial prototypes.^73^ Techniques such as structured data sharing and the integration of AI for data analysis and sharing could further enhance efficiency and reproducibility, even in the face of intellectual property concerns.^74^

Next, we discuss some strategies to tackle these challenges using enhanced protocols adapted from sister technologies (Figure 5c,d).

### Absorbing Successful Experience from Sister Technologies

The pathway of water electrolysis from innovation to market offers valuable lessons for CO_2_E development. We list some examples next.

#### Materials Design and MEAs

The PEM-based electrolyzer minimizes the loading of precious metals (<0.2 mg of iridium cm^–2^).^75^ Under alkaline conditions, non-noble NiFe layer doubled hydroxide (LDH) is a promising anode catalyst,^76^ also promising in CO_2_ electrolyzers. Ultrathin and selective membranes have enabled high energy efficiency in water electrolysis.^77−79^ The development of MEAs is critical for efficient CO_2_E due to their high surface area for catalytic reactions, facilitating reactant transport and minimizing ohmic losses.^80,81^

#### Full-Balance Analyses and Integration

While water electrolysis is highly selective (FE of 100%) and has high product purity (99.999%, H_2_),^82^ CO_2_E faces challenges related to complex input and output streams. Evaluating these streams, CO_2_ conversion rate, carbon efficiency, water use, and full-balance analyses including carbonate formation and product crossover is important for understanding CO_2_E systems.^83^

Different applications might rely on different CO_2_ sources that could impact input streams. For example, the direct use of diluted CO_2_ streams from point emitters may offer cost advantages but has to deal with the presence of sulfur and nitrogen oxides contaminants.^84^ At the downstream level, different product mixes with non-ideal FEs may be suitable depending on the target applications. Other integration examples, such as CO_2_E integrated into an MTO plant, would require specific conditions related to the output pressure of CO_2_ for efficient operation.^85^

#### Scaling up

CO_2_ electrolyzers can be improved and scaled up by learning from water electrolysis, using shared resources to lower costs.^86^ Enhancing efficiency through advanced techniques and using automated control for better monitoring and adjustments increase reliability. Custom programmable logic controllers (PLCs) are key for personalized control, integrating renewable energy, maximizing energy efficiency, and monitoring in real time.

## Technology Readiness Level (TRL) and Energy Consumption

**Figure 6:**
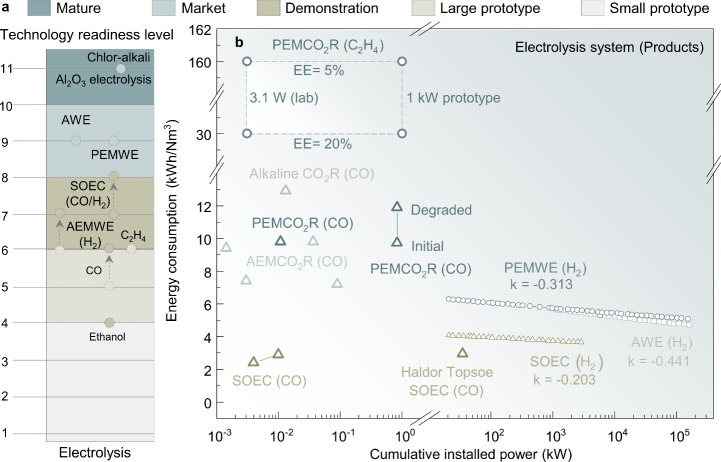
Comparison of technology readiness levels (TRLs) for major electrolysis technologies, including the chlor-alkali industry, electrolysis of water, and carbon dioxide reduction technology. TRLs are explained in Table S5.^87^ The energy consumption (kWh/Nm^3^) of carbon dioxide reduction at different scales, using water electrolysis as a reference.^61,94−100^ The analysis highlights the potential for reducing energy consumption as carbon dioxide reduction technologies move toward commercialization.

Electrolysis technologies have made significant progress in the last decades (Figure 6a). Alkaline water electrolysis (AWE) and PEMWE have reached high maturity (TRL = 9).^87^ Most low-temperature CO_2_E systems for the production of C_1_, C_2_ products are in large prototype stages, while SOEC systems have been in demonstration for the production of CO.^22^

High energy consumption stands as a prominent obstacle in the path toward large-scale CO_2_E implementation. The energy efficiency of high-temperature SOECs, up to 95%, is markedly higher than that of low-temperature water electrolysis.^88^ However, its current scale lags behind.^87^ The statical scaling slopes of SOECs (Figure 6b) are estimated to be −0.20 kWh/Nm^3^ per order of power, ranging from 1 kW to 100 MW. A corresponding decrease in energy consumption per magnitude indicates improved energy efficiency at larger scales of power generation.^89^ This slope is notably lower than that of low-temperature electrolysis, highlighting the need of managing both electrical energy and heat consumption in SOECs during scale-up.

Conversely, the scale of low-temperature PEMWE and AWE is comparable, reaching capacities of up to 1.1 GW and 1.4 GW, respectively.^87^ Both demonstrate similar high energy consumption levels (Figure 6b), approximately 5.74 kWh/Nm^3^ at the 1 MW scale.

However, with a significant increase to 100 MW, the energy consumption is reduced to 4.78–5.14 kWh/Nm^3^. The scaling slopes of PEMWE and AWE are estimated as −0.31 and −0.44 kWh/Nm^3^ per order of power from 1 kW to 100 MW, respectively, suggesting a slightly faster reduction in energy consumption for AWE, although both technologies are projected to have similar energy consumption levels.^89^ This highlights that the rules governing energy consumption reduction in different CO_2_E systems may vary during the scale-up process.

Processes with higher selectivity may require specific conditions or catalysts that impact energy consumption. Balancing these factors is crucial for optimizing the overall performance of CO_2_E systems.

It is important to note that different applications may have distinct requirements. For instance, if CO is intended for use as a Fischer–Tropsch input gas, the presence of some residual H_2_ in the CO flow might not be problematic. Simultaneous production of ethylene and ethanol may also be acceptable, as they are valuable chemicals and separate into gas and liquid phases without the need of additional separation costs. In such cases, achieving 100% selectivity may be less crucial, and the emphasis could be on minimizing energy consumption instead.^33^

The energy consumption of CO_2_E to CO utilizing AEM and PEM technologies is ca. 30% lower than that of alkaline CO_2_E below 100 W, due to the higher intrinsic energy efficiency of AEM- and PEM-based CO_2_E.^89^ Siemens and Evonik jointly demonstrated a 25 kW CO_2_R in a liquid flow cell,^90^ and Twelve also made a 2 kW bicarbonate electrolytic cell.^26^ The prospect of further scale-up stands to gain significantly from advancements in selectivity, stability, and energy efficiency.

The experience from water electrolysis suggests that (i) SOEC represents a viable technology for CO_2_E toward CO with improved energy efficiency, potentially enabling large-scale CO_2_E to the 10–100 MW level. (ii) In CO_2_E, the application scenarios for PEMWE and AWE can be determined based on their energy consumption characteristics. (iii) Enhancing selectivity is a prerequisite before considering scaling up.

Introducing new technologies like CO_2_E into markets dominated by economy-of-scale, large-volume industries such as the petrochemical industry is challenging. Key entry barriers include high initial capital expenditures, uncertain supply chains, and the necessity for substantial investments to benefit from economy-of-scale reduced costs.^91^ Securing funding for these demonstration projects is often difficult due to perceived risks associated with unproven technology and the absence of a clear path to commercialization. Venture capitalists, priming investment on companies that generate revenue from the expected rise of their value, may hesitate to invest in projects requiring substantial upfront capital without a well-defined pathway to profitability. Clear regulatory frameworks and carbon-related incentives could accelerate CO_2_E implementation. Additionally, it is important to consider alternative competing technologies, such as indirect CO_2_ reduction through clean hydrogen to produce synthetic fuels^92^ and biomass and bioenergy^93^ that generate energy from organic waste.

CO_2_E offers a way to displace fossil fuels in fuel and chemical feedstock production, leading to a net reduction in greenhouse gas emissions. The history of scaling up CO_2_ electrolysis shows a delay from lab to market that should be accelerated to this end.

We discuss strategies to catalyze this transition, bridging KPIs between laboratory and industrial scales. We suggest the critical value and potential of continuous TEA and LCA models informed from existing efforts at different increasing scales and the opportunities to leverage cumulative learnings during the scale-up of related technologies, especially water electrolysis. These encourage the implementation of standardization and certification protocols, adapted to CO_2_E technologies as a critical vector.

As CO_2_E technologies scale up, monitoring the energy consumption and efficiency of the different parts of the process is crucial to predict cost-competitive CO_2_E. In all cases, the implementation of CO_2_E technologies (single or in tandem) at increasing scales is crucial to identify and advance potential bottlenecks in supply and enabling technologies.

## Scale Requirements for Industrialization of CO_2_E

To achieve significant commercialization of CO_2_E, it is vital to determine the scale required to meet a portion of the market demand for various chemicals and fuels. Replacing just 10% of these products would require extensive electrolysis capacity, as the demand for carbon-based fuels and chemicals remains substantial (e.g., jet fuel and ethylene exceeds 300 million tons and 165 million tons, respectively). To achieve a 10% penetration in these markets, the scale of CO_2_ electrolysis systems must reach the hundreds of the MW scale, considering conversion efficiencies.

For CO_2_ to CO conversion, efforts like Haldor Topsoe’s eCOs technology are setting a benchmark with plants reaching a capacity of 96 Nm^3^ h^–1^ of CO. Achieving a substantial market impact would require many of such installations at increasing scales. To bridge this gap, progress in technology, integration, supply chains, and stakeholder engagement will be crucial.

## Supporting Information Available

Applications and synthesis methods of common chemicals, end-products’ market overview and carbon emissions, global CO_2_ evasion potential, CO_2_ reduction reactions to the desired products, merits of CO_2_E scaled-up pilots, and TRL explanation (PDF)
