# Supplementary material for: CO2 Electrolysis Technologies: Bridging the Gap toward Scale-up and Commercialization
Source: ACS Energy Lett. 2024 Aug 9;9(9):4293–305. doi: 10.1021/acsenergylett.4c00955 (PMC11406523; doi:10.1021/acsenergylett.4c00955)
Supplement: Supplementary file 1 — nz4c00955_si_001.pdf [file nz4c00955_si_001.pdf]

CO_2 electrolysis technologies: bridging the gap towards scale-up and commercialization

Blanca Belsa[1,2], Lu Xia[1,2] and F. Pelayo García de Arquer[1*]

[1]ICFO - Institut de Ciències Fotòniques, The Barcelona Institute of Science and Technology, Castelldefels (Barcelona), 08860, Spain

[2]These authors contributed equally: Blanca Belsa, Lu Xia

[*]corresponding author: pelayo.garciadearquer@icfo.eu

Summary of Contents:

- **S1. Chemical industry overview**
- **S2. Global CO_2 evasion potential**
  - CO_2 from current industrial production reliant on fossil fuels
  - CO_2 for CO_2E feedstock
  - KPIs from companies
- **S3. Technology Readiness Level (TRL)**

S1. Chemical industry overview

Table S1 | Uses and synthesis methods of common chemicals

Chemical	Use for/as	Current methods
CO	Acetic acid and phosgene production	Typically synthesized through the partial oxidation of carbon-containing compounds or as a by-product of other chemical reactions.
HCOOH	Preservative, antibacterial agent, and coagulant in various industries	Typically synthesized through the reaction of methanol and carbon monoxide under high pressure and temperature
MeOH	Chemical, fuel, solvent, pharmaceutical	Primarily synthesized from natural gas or biomass conversion processes.
EtOH	Food, beverage, pharmaceutical, chemical and fuel fields, including food additives, solvents, disinfectants and biofuels, etc.	Mainly by converting glucose through fermentation using yeast, or converting ethylene through chemical synthesis.
C_2H_4	Plastics, synthetic resins, lubricants, and fuels	Primarily through petroleum refining or cracking, and also through chemical reactions from ethane
Jet Fuel	Energy sources	Complex refining processes involving the distillation and treatment of crude oil to meet specific industry standards

Table S2 | End-products' market overview and carbon emissions.

Chemical	Market Size [billion USD]	Production [$Mt\cdot year^{-1}$]	Carbon footprint [$ton_{CO2eq}\cdot ton^{-1}{}_{product}$]	CO_2 emissions [$Mt\cdot year^{-1}$]	CO_2 feedstock for CO_2E [$Mt\cdot year^{-1}$]	Ref.
CO	5.7	8.1	-	4.6	12.7	[1,2]
HCOOH	0.8	1.0	2.2	2.2	1.0	[3–5]
MeOH	30.7	98.0	3.1	300.0	134.6	[6–8]
EtOH	82.0	110.0	1.0	110.0	210.2	[9–11]
C_2H_4	134.2	165.0	1.5	247.5	517.2	[12,13]

Jet Fuel	187.8	300.0	0.5	150.0	471.4	[14–16]

S2. Global CO_2 evasion potential

We conducted an estimation of the global CO_2 evasion potential by fully replacing fossil fuels with CO_2E in the production of key chemicals within the petrochemical and fuel industries. he chemicals targeted for replacement were ethylene (C_2H_4), ethanol (C_2H_5OH), methanol (CH_3OH), carbon monoxide (CO), formic acid (HCOOH) and Jet fuel. This estimation represents an upper bound, considering two significant factors. The first factor considers the global CO_2 emissions that would be avoided by discontinuing the current industrial output reliant on fossil fuels. The second factor accounts for the CO_2 consumed as a feedstock in the process of utilizing CO_2E. Together, these factors contribute to the maximum potential of CO_2 evasion resulting from the complete replacement of fossil fuels with CO_2E in the production of key chemicals within the petrochemical and fuel industries. It does not consider the carbon footprint associated with CO_2E and upstream (*e.g.*, Direct air capture or flue gas carbon capture) nor downstream technologies. It neither considers the emissions originated from combustion of such chemicals after their production

S1.1 <u>CO_2 from current industrial production reliant on fossil fuels</u>

The CO_2 emissions avoided from current industrial production is obtain by simply multiplying the current production volume by its Carbon footprint (Table S1). The obtained value is equal to the total CO_2 emissions associated with its actual production.

$$CO_2\ from\ current\ production\ [\mathrm{Mt}] = Annual\ production\ volume\ [\mathrm{Mt}] \times C\ footprint\ \left[\frac{\mathrm{kg\ CO_2\ emitted}}{\mathrm{kg\ product}}\right] \quad (1)$$

The data calculated was summarized in the **Table 1** of the main text.

S1.2 <u>CO_2 for CO_2E feedstock</u>

The CO_2 needed as a feedstock is obtained by multiplying the total annual production volume (Table S1) by the stoichiometric relationship from the corresponding electrochemical reduction reactions. An example calculation for ethylene is detailed below:

$$165\ \left[\frac{\mathrm{Mt\ C_2H_4}}{\mathrm{year}}\right] \times \frac{1\ \mathrm{mol\ C_2H_4}}{28.08\ \mathrm{g\ C_2H_4}} \times \frac{2\ \mathrm{mol\ CO_2}}{1\ \mathrm{mol\ C_2H_4}} \times \frac{44.01\ \mathrm{g\ CO_2}}{1\ \mathrm{mol\ CO_2}} = 517\ \frac{\mathrm{Mt\ CO_2}}{\mathrm{year}} \quad (2)$$

For jet fuel production, CO_2E serves as the source of CO, which is then subject to various technologies to undergo further upgrading. Consequently, the CO_2 utilized as feedstock for jet fuel is identical to the CO derived from CO_2E.

Table S3 | CO_2 reduction reactions to the desired products

$\mathrm{CO_2 + 2H^+ + 2e^- \rightarrow CO + H_2O}$	(3)
$\mathrm{CO_2 + 2H^+ + 2e^- \rightarrow HCOOH}$	(4)
$\mathrm{CO_2 + 6H^+ + 6e^- \rightarrow CH_3OH}$	(5)
$\mathrm{2CO_2 + 12H^+ + 12e^- \rightarrow C_2H_5OH + 3H_2O}$	(6)
$\mathrm{2CO_2 + 12H^+ + 12e^- \rightarrow C_2H_4 + 4H_2O}$	(7)

Table S4 | Figures of merit chart of CO_2E scaled-up pilots.

Company	Target Product	Device type	Stability [h]	Current density [$mA\cdot cm^{-2}$]	Faradaic Efficiency [%]	Cell Voltage [V]	Power [kW]	Surface Area [cm^2]	Capacity	Ref.
**Avantium**	HCOOH	GDE flow cell	4	200	98			10		[17]
**Avantium**	CO			200						[18]
**CERT Systems / UofT**	C_2H_4	MEA	100	100-400	50	3.6-4		5		[19]
**CERT Systems / UofT**	C_2H_4	MEA	*2,400 *cumulative				50	40000[a)]	*100 kg $CO_2\cdot day^{-1}$ *projected	[20–22]
**DNV**	HCOOH	Liquid Flow Cell	96	40	60			600	1 kg $CO_2\cdot day^{-1}$	[23]
**Dioxide Materials**	CO	MEA	760	120	98	2.8-3.0		250		[24]
**Dioxide Materials**	HCOOH	MEA	500	200	65-70	3.5		5		[25]
**Dioxide Materials**	HCOOH	MEA	1,000	200	73-91	3.52		5		[26]
**Haldor Topsoe**	CO	SOEC	2,000	450	100	1.24	2.96 kW Nm^{-3}		12 $Nm^3\cdot h^{-1}$	[27,28]
**OCOChem**	HCOOH							100		
**Prometheus**	EtOH	MEA			50				9 kt of atm $CO_2\cdot year^{-1}$	[29]
**Siemens and Evonik**	CO	Liquid Flow Cell	1,200	300	96	7-7.5		10		[30]

**Siemens and Evonik**	CO	Flow Cell	200	150	52	8.11-6.17	52.65 kWh	75	3.6 $L \cdot h^{-1}$	[31]
**Siemens and Evonik**	CO	Flow Cell					25	3,000$^{b)}$		[32,33]
**Siemens and Evonik**	C_2H_4	Flow Cell	168		70					[34]
**Siemens and Evonik**	C_2H_4	PEM	6	150-200		12		300		[35]
**Sunfire**	CO	SOEC							730 kg $CO_2 \cdot h^{-1}$	[36]
**Twelve**	CO	PEM MEA stack					2		5 kg $CO_2 \cdot day^{-1}$	[37]
**Twelve**	CH_4	PEM MEA stack			97					[37]
**Twelve**	C_2H_4	PEM MEA stack								[37]
**eChemicles**	CO	MEA cell	2,000	300	90	~2.8		8		[38]
**Carbon Energy Technology**	Syngas	MEA cell	2,000	50-150	30-95	2.7-3.0		2500		[39]

The data has been sourced from a combination of industry reports, white papers, and publicly available company documents, along with academic publications where available. This blend of sources provides a comprehensive view, though it's important to note the varying levels of peer review and formal validation among these documents.

S3. Technology Readiness Level (TRL)

Table S5 | Technology Readiness Level (TRL), incorporating the commonly used TRL grading standards from National Aeronautics and Space Administration (NASA) and the US Department of Defense (DoD), and also the TRLs for electrolyzers from the International Energy Agency.[40,41]

TRL	Explanation
TRL-1	Basic principles: Technology concept has been formulated, but it is yet to be experimentally verified.
TRL-2	Technology concept validation: Feasibility of the technology has been shown through laboratory studies.
TRL-3	Proof of concept: Initial experimentation confirms the technology's viability in a laboratory environment.
TRL-4	Component and subsystem validation: The technology has been tested in a laboratory setting, demonstrating its functionality in simulated conditions.
TRL-5	Validation in relevant environment: The technology has been tested in a relevant environment, displaying its basic performance.
TRL-6	System model or prototype demonstration: The technology has been showcased in a relevant operational environment, exhibiting complete performance.
TRL-7	System prototype demonstration: The technology has been proven to be effective and practical through testing in an operational system.
TRL-8	System complete and qualified: The technology has been proven to be reliable, stable, and effective under operational conditions.
TRL-9	Actual system proven in operational environment: The technology has been successfully demonstrated in actual applications and is ready for commercial use.
TRL-10	Large-scale commercially proven.
TRL-11	Huge market success and classic technology.

References

1. Global Carbon Monoxide Industry – Industry Reports. https://www.360researchreports.com/global-carbon-monoxide-industry-18194935. Date accessed: 14/12/2021

2. De Luna, P. et al. What would it take for renewably powered electrosynthesis to displace petrochemical processes? *Science* **364**, 6438 (2019).

3. Rumayor, M., Dominguez-Ramos, A. & Irabien, A. Formic Acid Manufacture: Carbon Dioxide Utilization Alternatives. *Appl. Sci.* **8** (6), 914 (2018).

4. Global Formic Acid Market – 360 Market Updates. https://www.360marketupdates.com/global-formic-acid-market-14199138. Date accessed: 14/12/2021

5. Formic Acid Market Size, Share, Growth | Report, 2030. https://www.marketresearchfuture.com/reports/formic-acid-market-1132. Date accessed: 14/12/2021

6. Methanol Market Global Forecast to 2026 | MarketsandMarkets. https://www.marketsandmarkets.com/Market-Reports/methanol-market-425.html. Date accessed: 14/12/2021

7. Methanol Market Size, Share & Trends | Analysis Report [2028]. https://www.fortunebusinessinsights.com/industry-reports/methanol-market-101552. Date accessed: 14/12/2021

8. Irena & Methanol Institute. Innovation Outlook: Renewable Methanol. /publications/2021/Jan/Innovation-Outlook-Renewable-Methanol (2021). Date accessed: 14/12/2021

9. Ethanol Market Size, Share, Growth | Report, 2030. https://www.marketresearchfuture.com/reports/ethanol-market-7304. Date accessed: 14/12/2021

10. Annual Ethanol Production. https://ethanolrfa.org/markets-and-statistics/annual-ethanol-production. Date accessed: 14/12/2021

11. Carbon Dioxide - Attis Biofuels. https://www.attisbiofuels.com/by-products/carbon-dioxide. Date accessed: 22/12/2021

12. Alex Tullo. The search for greener ethylene. C&EN Global Enterprise 99, 20–22 (2021).

13. Ethylene Market Size, Share, Growth | Report, 2030. https://www.marketresearchfuture.com/reports/ethylene-market-931. Date accessed: 14/12/2021

14. Ng, K. S., Farooq, D. & Yang, A. Global biorenewable development strategies for sustainable aviation fuel production. *Renew. Sustain. Energy Rev.* **150**, 111502 (2021).

15. Commercial airlines: worldwide fuel consumption 2023 | Statista. https://www.statista.com/statistics/655057/fuel-consumption-of-airlines-worldwide/. Date accessed: 01/08/2023

16. Jet Fuel Market Statistical Analysis and Growth Forecast - 2031. https://www.alliedmarketresearch.com/jet-fuel-market-A06883. Date accessed: 01/08/2023

17. Pavesi, D. *et al.* Cathodic disintegration as an easily scalable method for the production of Sn-and Pb-based catalysts for CO_2 reduction. *ACS Sustain. Chem. Eng.* **8**, 15603–15610 (2020).

18. Gruter, G.-J. *Innovation Management (choosing options) both from Industrial and Academic Perspective- PART 2 (2020)*. http://www.elcorel.org/files/Aventium/GRUTER%20Avantium%20ELCOREL%20INNOVATION%20MANAGEMENT%2014%20Oct%202020%20PART%202.pdf. Date accessed: 22/12/2021

19. Gabardo, C. M. *et al.* Continuous carbon dioxide electroreduction to concentrated multi-carbon products using a membrane electrode assembly. *Joule* **3**, 2777–2791 (2019).

20. Edwards, J. P. *et al.* Pilot-Scale CO_2 electrolysis enables a semi-empirical electrolyzer model. *ACS Energy Lett* **8**, 2576–2584 (2023).

21. CERT | Carbon XPRIZE. https://carbon.xprize.org/prizes/carbon/teams/cert. Date accessed: 05/01/2022

22. Toronto-based XPRIZE Finalist shows how to turn carbon emissions into renewable fuel. https://taf.ca/toronto-xprize-finalist-turn-carbon-emissions-renewable-fuel/. Date accessed: 05/01/2022

23. Det Norkse Veritas (DNV). Carbon dioxide utilization electrochemical conversion of CO_2-Opportunities and challenges. *Research and Innovation, Position Paper 07* (2011).

24. Kaczur, J. J., Yang, H., Liu, Z., Sajjad, S. D. & Masel, R. I. A Review of the use of immobilized ionic liquids in the electrochemical conversion of CO_2. *C* **6**, 33 (2020).

25. CO_2 conversion to formic acid | Dioxide Materials. https://dioxidematerials.com/technology/formic-acid/. Date accessed: 14/12/2021

26. Yang, H., Kaczur, J. J., Dawar Sajjad, S. & Masel, R. I. Performance and long-term stability of CO_2 conversion to formic acid using a three-compartment electrolyzer design. *Journal of CO_2 Utilization* **42**, 2212–9820 (2020).

27. Haldor Topsoe. Small-Scale CO from CO_2 using electrolysis. *Chemical Engineering World* 44–46 (2017).

28. Küngas, R. Review—Electrochemical CO_2 reduction for CO production: Comparison of low- and high-temperature electrolysis technologies. *J Electrochem. Soc.* **167**, 044508 (2020).

29. Prometheus Fuels - Technology. https://prometheusfuels.com/technology. Date accessed: 14/12/2021

30. Haas, T., Krause, R., Weber, R., Demler, M. & Schmid, G. Technical photosynthesis involving CO_2 electrolysis and fermentation. *Nat. Catal.* **1**, 32–39 (2018).

31. Jeanty, P. *et al.* Upscaling and continuous operation of electrochemical CO_2 to CO conversion in aqueous solutions on silver gas diffusion electrodes. *Journal of CO_2 Utilization* **24**, 454–462 (2018).

32. Rheticus: World's-first-automated-CO2-electrolyzer | 2020 | Siemens Energy Global. https://www.siemens-energy.com/global/en/news/magazine/2020/rheticus-worlds-first-automated-co2-electrolyzer.html. Date accessed: 21/03/2022

33. The climate killer becomes a raw material | Magazines | Siemens Energy Germany. https://www.siemens-energy.com/de/de/news/magazine/anlage-nutzt-co2-fuer-kuenstliche-photosynthese.html. Date accessed: 14/06/2023

34. Kuhn, K.-J. *Electrolysis / eFuels Pathways to synthetic eFuels*. (2020). Siemens Energy. https://www.wko.at/oe/oegew/co-electrolysis-and-synthesis.pdf. Date accessed: 16/06/2023

35. Roth, A. *et al. Type Deliverable 6.8: Demonstration on plant testing, compiling of simulation and hardware results. CO_2-based Electrosynthesis of Ethylene oXIDE* (2021) | Grant Agreement No.: 768789 (H2020-SPIRE-2017).

36. Sunfire. Renewable syngas for e-fuel and chemicals production. https://www.sunfire.de/en/about-us (2021). Date accessed: 14/12/2021

37. SoCalGas RD&D & Opus 12 Present: "Utilization of CO2 Emissions to Make Renewable Fuels & Chemicals" - YouTube. https://www.youtube.com/watch?app=desktop&v=ZLSltwMp3Vs. Date accessed: 22/12/2021

38. Raya-Imbernón, A. et al. Renewable syngas generation via low-temperature electrolysis: Opportunities and challenges. *ACS Energy Lett* **9**, 288–297 (2024)

39. Cheng, Y., Hou, P., Wang, X. & Kang, P. CO_2 electrolysis system under industrially relevant conditions. *Acc. Chem. Res.* **55**, 231–240 (2022).

40. IEA. *Electrolysers*. https://www.iea.org/reports/electrolysers (2022). Date accessed: 16/05/2023

41. Héder, M. From NASA to EU: the evolution of the TRL scale in Public Sector Innovation. *Innov J* **22**, 3.
